# Ser77Tyr transthyretin amyloidosis in Israel: Initial manifestations and diagnostic features

**DOI:** 10.1002/acn3.51741

**Published:** 2023-02-11

**Authors:** Amir Dori, Michael Arad, Yishay Wasserstrum, Arthur Pollak, Vera Nikitin, Merav Ben‐David, Jana Shamash, Ayelet Hashachar Nahum, Efrat Shavit‐Stein, Liran Domachevsky, Rafael Kuperstein, Dan Dominissini, Natalia Shelestovich, Menachem Sadeh, Elon Pras, Lior Greenbaum

**Affiliations:** ^1^ Department of Neurology Sheba Medical Center Tel Hashomer Israel; ^2^ Sackler Faculty of Medicine Tel Aviv University Tel Aviv Israel; ^3^ The Leviev Heart Center, Sheba Medical Center Tel Hashomer Israel; ^4^ Department of Cardiology, Hadassah Medical Center Hebrew University of Jerusalem Jerusalem Israel; ^5^ The Danek Gertner Institute of Human Genetics, Sheba Medical Center Tel Hashomer Israel; ^6^ Department of Nuclear Medicine Sheba Medical Center Tel Hashomer Israel; ^7^ The genomics Unit Sheba Cancer Research Center, Sheba Medical Center Tel Hashomer Israel; ^8^ Wohl Institute of Translational Medicine, Sheba Medical Center Tel Hashomer Israel; ^9^ Department of Pathology Sheba Medical Center Tel Hashomer Ramat Gan Israel; ^10^ Department of Neurology Wolfson Medical Center Holon Israel; ^11^ The Joseph Sagol Neuroscience Center, Sheba Medical Center Tel Hashomer Israel

## Abstract

**Objective:**

Amyloidosis due to the transthyretin Ser77Tyr mutation (ATTRS77Y) is a rare autosomal‐dominant disorder, characterized by carpal‐tunnel syndrome, poly‐ and autonomic‐neuropathy, and cardiomyopathy. However, related symptoms and signs are often nonspecific and confirmatory tests are required. We describe the age and frequency of early symptoms and diagnostic features among individuals of Jewish Yemenite descent in Israel.

**Methods:**

Records of mutation carriers were retrospectively reviewed. ATTRS77Y diagnosis was defined by the presence of amyloid in tissue and/or amyloid‐related cardiomyopathy.

**Results:**

We identified the Ser77Tyr mutation at the heterozygous state in 19 amyloidosis patients (mean age at diagnosis: 62 ± 5.7 years, range 49–70) and 30 amyloid‐negative carriers. The probability for disease diagnosis increased from 4.4% at age 49 to 100% at 70 and occurred earlier in males. Initial symptoms preceded diagnosis by 5 ± 3.8 years (range 0–12) and were commonly sensory changes in the extremities. Erectile dysfunction predated these in 8/13 (62%) males. In two patients cardiac preceded neurological symptoms. Two patients declined symptoms. Electrophysiological studies near the time of diagnosis indicated a median neuropathy at the wrist in 18/19 (95%) and polyneuropathy in 13/19 (68%). Skin biopsy revealed epidermal denervation in 15/16 (94%) patients. Cardiomyopathy was identified in 16/19 (84%). Sensory complaints or epidermal denervations were present in 17/30 (57%) of amyloid‐negative carriers and co‐occurred in 10/30 (33%).

**Interpretation:**

ATTRS77Y symptoms commonly occur after age 50, but may begin earlier. Median neuropathy, skin denervation and cardiomyopathy are frequently identified. Symptoms may be absent in patients and common in amyloid‐negative carriers.

## Introduction

Pathogenic mutations in the transthyretin (*TTR*) gene cause variant TTR amyloidosis (ATTRv), inherited in an autosomal dominant manner. ATTRv is a severe but treatable multisystem disorder, characterized by a combination of entrapment‐, autonomic‐ and poly‐neuropathy, cardiomyopathy, gastrointestinal and renal disease as well as ocular, leptomeningeal and brain involvement.^1^, ^2^ Early treatment with recently approved drugs can delay or prevent disease progression.^3^, ^4^, ^5^, ^6^, ^7^ More than 150 mutations were identified in *TTR*. Some show a genotype–phenotype correlation, with predominant or initial neurological or cardiological manifestations, consistent with amyloid accumulation mainly in peripheral nerves or heart.^8^

Suspicion of disease initiation and pro‐active pursuit of diagnosis is largely based on data about the genotype‐associated features, age at disease onset, and penetrance. Several *TTR* mutations are endemic in particular populations.^9^ In Israel, amyloidosis due to the Ser77Tyr mutation (ATTRS77Y) was previously described among families of Jewish Yemenite descent.^10^, ^11^ ATTRS77Y is associated with a late‐onset progressive polyneuropathy, frequent carpal tunnel syndrome (CTS), and cardiac disease.^8^ Manifestation of related symptoms or signs in a Ser77Tyr mutation carrier should therefore raise suspicion of amyloidosis.^2^ However, resembling nonspecific symptoms due to neuropathy are common in the general population, and attributing them specifically to ATTRS77Y is not straightforward. Furthermore, this may be particularly challenging in the presence of additional disorders that are associated with risk of polyneuropathy and CTS such as type 2 diabetes mellitus (T2D). In these cases, the presence of amyloid in tissue or typical amyloid‐related cardiac involvement is key evidence for amyloidosis and the need for treatment.

Skin punch biopsy became an attractive tool for confirming the diagnosis of amyloidosis. Deposition of amyloid in skin correlates with worsening small nerve fiber pathology and severity of neuropathy symptoms and staging.^12^, ^13^ The sensitivity of a punch skin biopsy for detecting amyloid in individuals with ATTRv was reported to be high when collected from both the ankle region and thigh,^13^ and the highest deposition rate was reported at the ankle region.^12^ Accordingly, two skin biopsies from the ankle region may provide the best sensitivity for detecting amyloid.

Echocardiography is commonly used to screen for increased left ventricular hypertrophy (LVH), which is typical for cardiac amyloidosis. When present, amyloidosis may be confirmed and specified by identifying amyloid deposits within an endomyocardial or an extra‐cardiac biopsy.^14^ In the absence of histology, cardiac ATTRv can be diagnosed by bone scintigraphy employing ^99m^Tc‐pyrophosphate (PYP), ^99m^Tc‐3,3‐diphosphono‐1,2‐propano‐ dicarboxylic acid (DPD) or ^99m^Tc‐hydroxymethylene diphosphonate if a monoclonal gammopathy is excluded.^15^ However, scintigraphy shows low sensitivity in some TTR mutations including Ser77Tyr.^16^, ^17^ In these cases, cardiac magnetic resonance (CMR) imaging may be used to show characteristic amyloidosis findings.^16^, ^18^

In this study, we employed tissue biopsies, most commonly of skin, and/or cardiac criteria for diagnosis of ATTR in Ser77Tyr mutation carriers. The age and frequency of early symptoms, and of clinical, electrophysiological, and cardiac findings at the time of diagnosis in 19 ATTRS77Y patients were determined. We also describe the characteristics of 30 amyloid‐negative carriers and estimated the age of disease onset according to the age at diagnosis.

## Methods

### Patients and clinical assessment

We retrospectively reviewed medical records of all *TTR* Ser77Tyr mutation carriers evaluated at the Sheba Medical Center (SMC) neuromuscular clinic, a tertiary referral center in Israel, between January 2016 and December 2022. The study was approved by the institutional review board (7476‐20‐SMC and 7238‐20‐SMC).

All participants underwent a detailed neurological examination. Sensory, motor, autonomic, and cardiac‐related symptoms and signs were collected.^19^ Muscle strength was scored according to the Medical Research Council (MRC) scale and lower limbs according to the neuropathy impairment scale (NIS‐LL).^20^ Ambulation was scored according to familial amyloid polyneuropathy (FAP) and polyneuropathy disability (PND) scores.^21^ Laboratory evaluation for detection of systemic disorders that are associated with neuropathy included the following: complete blood count, electrolyte profile, hemoglobin A1c, vitamin B12 and folic acid levels, thyroid, renal and liver function tests, immunofixation electrophoresis and immunoglobulin and light‐chain quantification, antinuclear antibody, rheumatic factor, SS‐A, SS‐B, complement 3 and 4, transglutaminase and C‐reactive protein levels. Urine was tested for electrolytes, glucose, and protein and employed Bence Jones/immunofixation assays.

### Mutation screening

All patients gave informed consent for genetic testing. Some of the patients underwent sequencing of the entire *TTR* gene coding region by commercial laboratories: Centogene (Germany) or Genetic Medico‐diagnostic laboratory Genica and Genome Center (Bulgaria). For the rest, targeted Sanger sequencing of the Ser77Tyr mutation in the *TTR* gene was performed at SMC or Hadassah Medical Center in Israel.

### Electrophysiological studies

Nerve conduction studies (NCS) and electromyography (EMG) were performed at SMC as previously described.^22^ Electrophysiological studies were mandatory for all carriers at age 45 or above or earlier if sensory symptoms were reported. Motor NCSs included median, ulnar, tibial and peroneal nerves (Table S1). Sensory NCSs included median, ulnar, radial, sural and superficial peroneal nerves (Table S2). Bilateral upper limbs were tested in all but one case. Orthodromic median mixed‐nerve studies across the wrist were performed when the median sensory study was normal. In the lower limbs, motor studies were conducted on one side and sensory studies bilaterally. The sympathetic skin response (SSR) was tested in the palm and foot on one side when the sural study was normal or near‐normal. EMG was performed on one lower extremity and included but was not limited to the tibialis anterior, medial gastrocnemius and quadriceps vastus medialis.

### Cardiovascular evaluation

Cardiac evaluation included echocardiography when complaints were suspected as cardiac‐related and for all carriers above age 45. Heart failure symptoms were classified according to the New York Heart Association (NYHA).^23^ Cardiac involvement was defined by left ventricular hypertrophy (LVH) with interventricular septum thickness ≥12 mm by transthoracic echocardiography, in the presence of a Congo red positive biopsy. When LVH was present but biopsies were not available or negative for Congo red staining, a positive bone scintigraphy (with ^99m^Tc‐PYP or ^99m^Tc‐DPD) or typical CMR^16^ in the absence of a monoclonal gammopathy was required to confirm the diagnosis of ATTR. When echocardiography was negative, scintigraphy and/or CMR were employed if clinical suspicion was high, or when heart failure or an alternative cause for hypertrophy was present.

### Histopathology

Skin biopsies (3.0‐mm punch) were performed at the distal leg 10 cm proximal to the lateral malleolus (ankle) using standard procedures.^24^ Intraepidermal nerve fiber density (IENFD) quantitation in 50 μm thick floating sections^24^ employed immunofluorescence microscopy against PGP9.5. Normal IENFD values were based on published data^25^ adjusted according to controls at SMC. IENFD below the 5^th^ percentile for age was determined abnormal, indicating small‐fiber polyneuropathy.

Congo red staining was performed on at least 5 additional 16 μm glass‐mounted sections obtained from the same biopsy used for IENFD analysis. Sections were lightly counterstained with hematoxylin. Amyloid deposition was identified by autofluorescence^26^ followed by light microscopy to confirm dark red to yellow‐green birefringence of plaques under polarized light.^27^

### Definition of ATTRS77Y patients versus amyloid‐negative mutation carriers

*TTR* Ser77Tyr mutation carriers were suspected to be affected by amyloidosis if a related objective sign, or symptom accompanied by an abnormal test was present.^19^ However, diagnosis of ATTR (i.e. ATTR patients) required confirmation by histopathological evidence for amyloid^28^, ^29^ and/or identification of cardiomyopathy consistent with amyloidosis. Skin biopsy, which is a sensitive tool to detect amyloid deposits at ATTR polyneuropathy disease even at disease onset^12^, ^13^ was most commonly used. Age at ATTR diagnosis was defined according to the earliest confirmatory test (biopsy or scintigraphy/CMR). The delay from symptom onset was recorded. When a free light chain abnormality was detected in the serum or urine, the presence of TTR in tissue biopsy was confirmed and light‐chain related amyloidosis was excluded by immunohistochemistry and/or western‐immunoblot typing.^30^

Carriers were determined as amyloid‐negative if (1) two skin biopsies were Congo‐red negative and (2) echocardiography ruled‐out LVH in individuals above age 45 or when cardiac‐related symptoms were suspected at any age. If LVH was present, CMR or scintigraphy was indicated and had to be negative as well.

### Statistical analysis

Patient characteristics are reported as mean ± standard deviation and range. Differences between independent groups were calculated by independent *t*‐test. Fisher Exact test was conducted to compare the risk of disease between age groups. The probability of becoming diagnosed with increasing age was calculated by Kaplan–Meier survival analysis. Statistical analysis was performed using GraphPad Prism 9.

## Results

### *TTR* mutation screening

We identified the heterozygous *TTR* Ser77Tyr mutation in 56 subjects, all self‐reportedly descendants of 5 Jewish families that emigrated to Israel from Yemen. Evaluation sufficient to define subjects as amyloidosis patients or amyloid‐negative was available for 49 mutation carriers.

### Characterization of ATTRS77Y patients

#### Initial common clinical manifestations

Nineteen ATTRS77Y patients which fulfilled the diagnostic criteria for amyloidosis were identified (13 males; Tables 1 and 2). The mean age at diagnosis was 62 ± 5.7 (range 49–70) and was similar between families (62 ± 3.2). In 18/19 (95%) patients an extra‐cardiac biopsy was positive, of which two also had a positive endocardial biopsy which confirmed the diagnosis. One patient (case 2) had no biopsy available and in that case ATTR diagnosis was based on the presence of cardiomyopathy per echocardiography and typical CMR.

**Table 1:** Patient characteristics: demographics, age at symptom onset and on exams, exam scores, electrodiagnostic and biopsy findings.

Case	Sex	Family	Clinical	Symptoms		Exam scores					Electrophysiological studies						Biopsy		
			Additional disorders	Age at UE onset (y)	Age at LE onset (y)	Age at exam (y)	FAP	PND	MRC score	NIS‐LL	Age at NCS (y)	Median neuropathy: Bilateral	Median neuropathy:Unilateral	Polyneuropathy	LE fibrillations	SSR abnormality	Age at Biopsy (y)	IENFD <5^th^ percentile	Congo red positive tissue
1	M	A	Renal failure	‐	‐	49	0	0	60	2	49	−	+	−	NA	−	49	+	Skin
2	M	A	Liver transplant	63	57	61	1	1	60	4	68	+	−	+	+	NA	NA	NA	NA
3	M	A	T2D, Renal transplant	62	69	69	1	1	59	4	69	+	−	+	+	NA	66	NA	RC
4	M	B		58	57	57	1	2	45	11	58	+	−	+	+	NA	58	+	Skin
5	M	B	Smoldering myeloma	60	60	61	1	2	57	24	61	+	−	+	+	NA	61 61	+	EC, Skin
6	M	B	HG	‐	‐	63	0	0	58	0	63	+	−	+	−	−	63	+	Skin
7	F	C		46	51	57	1	1	60	0	58	+	−	−	−	−	58	−	Skin
8	F	C	T2D, MGUS	48	56	58	1	2	56	20	58	+	−	+	−	NA	58	+	Skin
9	M	C	T2D	54	NA	59	0	0	60	4	59	+	−	−	+	−	59	+	Skin
10	M	C	T2D	53	58	61	3	4	48	52	61	+	−	+	+	NA	62	+	Skin
11	M	C	HG	59	63	63	1	2	59	22	63	+	−	+	+	NA	63	+	Skin
12	M	C	DMT2D, RHD	NA	64	65	1	1	58	13	65	+	−	−	+	−	65	+	Skin
13	F	C	T2D	61	NA	65	1	1	60	0	66	+	−	−	−	−	66	+	Skin
14	M	D	PKD, renal transplant	53	53	52	1	1	60	0	53	−	−	−	−	−	52 53	+	EC, skin
15	M	D		55	55	57	1	1	58	14	57	+	−	+	+	NA	57	+	Skin
16	M	D	T2D	56	54	62	1	2	60	19	63	+	−	+	+	NA	63	+	Skin
17	F	D	T2D, MGUS	64	68	69	2	3b	52	51	70	+	−	+	+	NA	70	+	Skin
18	F	D	T2D	71	71	72	1	2	50	34	72	−	+	+	−	NA	70	NA	BM
19	F	E	T2D	57	65	67	1	2	60	5	68	+	−	+	NA	NA	68	+	Skin

BM, bone marrow; EC, endocard; F, female; FAP, familial amyloid polyneuropathy; HG, hyperglycemia; IENFD, intraepidermal nerve fiber density percentile; LE, lower extremity; M, male; MGUS, monoclonal gammopathy of undetermined significance; MRC, medical research council; NA, not applicable/applied; NCS, nerve conduction studies; NIS‐LL, neurology impairment score of lower limb; PKD, polycystic kidney disease; PND, polyneuropathy disability; RC, rectum; RHD, rheumatic heart disease; SSR, sympathetic skin response; T2D, type 2 diabetes; UE, upper extremity; y, years.

**Table 2:** Cardiac evaluation findings in patients.

Case	Cardiomyopathy	NYHA	Electrocradiogram				Echocardiography						CMR			Scintigraphy		
			Sinus rhythm	Conduction abnormalities	Arrhythmia	Low‐voltage	Age at evaluation (y)	IVS (mm)	PWD (mm)	LVEF%	GLS	GLS pattern (strain)	Age at evaluation (y)	Maximal wall thickness	LGE typical pattern	Age at evaluation (y)	Tracer	Perugini grade
1	−	1	+	Normal	None	−	51	11	10	60	−21	Normal						
2	+	NA	+	Normal	NSVT	−	60	17	17	45	−8.2	Compatible	65	23	+			
3	+	3	+	PRWP	PAF, PAT, PAC's	+	67	15	13	50	−8	Typical	69	25	+	68	DPD	1
4	+	3	+	Normal	AFL	−	58	13	10	60	−11.2	Typical						
5	+	NA	+	Q in V2	PVC's, NSVT	+	60	15	15	39	−6.8	Compatible	61	21	+	61	PYP	3
6	+	2	+	First degree AVB, Lt axis	None	−	63	14	13	55	−8.5	Typical	67	22	+	64	PYP	0
7	−	1	+	Normal	None	−	57	10	10	60								
8	+	NA	+	ICRBBB, LAHB	AFL	+	58	12	8	55	−6.9	Compatible				58	DPD	0
9	+	1	+	Lt axis dev, Q in V2		−	59	12	9	58	−14.4	Compatible				62	DPD	0
10	+	NA	+	LAHB, Precordial STE	None	−	60	16	9	50	−9.5	Compatible						
11	+	NA	+	PRWP, LAHB	PVC's	−	64	13	13	60								
12	+	2	+	LBBB	VPBs	−	65	13	13	35	−10	Atypical	66	19	+	66	PYP	1
13	−	2	+	Normal		−	66	9	8	60								
14	+	2	+	Normal		−	50	18	15	60	−12.8	Compatible	52	17	+	52	DPD	1
15	+	3	+	Normal	None	−	57	13	10	60	−12.4	Atypical						
16	+	2	+	LAHB	PVC's	−	63	16	13	35	−10	Typical	63	16	+	63	PYP	2
17	+	1	+	CRBBB	None	−	69	13	14	55								
18	+	3	+	PRWP	NSVT	+	67	9	10	30			70	10	+	70	DPD	1
19	+	3	−	RBBB, LAHB	Perm. AF	−	65	12	12	60	−11.5	Typical						

AF, atrial fibrillation; AFL, atrial flutter; AVB, atrioventricular block; CMR, Cardiac magnetic resonance; CRBBB, complete right bundle branch block; DPD, ^99m^Tc‐3,3‐diphosphono‐1,2‐propano‐ dicarboxylic acid; GLS, global longitudinal left ventricular strain; ICRBBB, incomplete right bundle branch block; IVS, Inter‐ventricular septum; LAHB, left anterior hemi‐block; LBBB, left bundle branch block; LGE, Late gadolinium enhancement (compatible with amyloidosis); LV, left ventricle; LVEF, left ventricular ejection fraction; NA, Not applicable; NYHA, New York Heart Association, NSVT, non‐sustained ventricular tachycardia; PAC's, premature ventricular contractions; PAF, paroxysmal atrial fibrillation; PAT, paroxysmal atrial tachycardia; Perm, permanent atrial fibrillation; PRWP, poor R‐wave progression; PVC's, premature ventricular contractions; PWD, Posterior wall diameter; PYP, ^99m^Tc‐pyrophosphate; RBBB, right bundle branch block; STE, ST‐segment elevation; VPBs, ventricular premature beats; y, years.

Initial symptoms appeared at the mean age of 57 ± 6.3 years (range 46–71), commonly with sensory complaints of tingling, numbness, pain or sensory loss in the hands in 13/19 (6%) patients (Table 1). These were probably related to CTS, according to NCSs that were performed in the following 5 ± 3.7 years (range 0‐12). Similar sensory symptoms in the feet followed those in the hands in 8/19 (42%), accompanied them in 4/19 (21%) and preceded them or where the only site of sensory disturbance in 3/19 (16%). In only one patient motor symptoms appeared prior to sensory complaints.

Erectile dysfunction predated these symptoms in the limbs in 8/13 (62%) males. Early gastrointestinal symptoms were reported by only one patient, but later in the course constipation was reported by 5/19 (26%), diarrhea by 4/19 (21%), and weight loss by 10/19 (53%) patients. Importantly, 2 patients (49‐ and 63‐year‐old males) had no complaints and declined any neuropathic, autonomic, or dysfunctional symptoms.

Neurological examination near the time of diagnosis showed a distal sensory loss in only about a third of patients: pain in 7/19 (37%), vibration in 8/19 (42%) and proprioception in 6/19 (32%). Distal weakness and absent Achilles deep tendon reflex were more common, present in 10/19 (53%) and 13/19 (68%) of patients, respectively.

Additional disorders which are associated with polyneuropathy were present in 16/19 (84%) patients. In all of them, these delayed evaluation and identification of amyloidosis. These included T2D or hyperglycemia in 12 subjects, and monoclonal gammopathy of undetermined significance (MGUS) in 2, which both also had T2D. One patient exhibited a smoldering myeloma on bone marrow biopsy. Three patients had renal failure, requiring hemodialysis in two followed by renal transplantation, related in one case to polycystic kidney disease.

#### Electrodiagnostic manifestations

Electrodiagnostic studies performed in proximity to diagnosis indicated a median neuropathy at the wrist in 18/19 (95%) patients, which was bilateral in 16/19 (84%; Table 1).

Sensory or sensory‐motor axonal polyneuropathy was evident in 13/19 (68%) patients, and EMG showed active denervation in 11/17 (65%), as indicated by lower extremity fibrillations. The 6 patients which showed no evidence of a large‐fiber polyneuropathy had normal SSR, providing no evidence for the presence of small‐fiber sympathetic neuropathy.

### Skin biopsy for assessment of small fiber neuropathy and tissue diagnosis of amyloidosis

Skin biopsy was performed by 16/19 patients at age 61 ± 5.5 (range 49–70); two samples were obtained in 11/16 cases. The IENFD was abnormally low, indicating epidermal denervation in 15/16 (94%) patients (Table 1).

**Figure 1:**
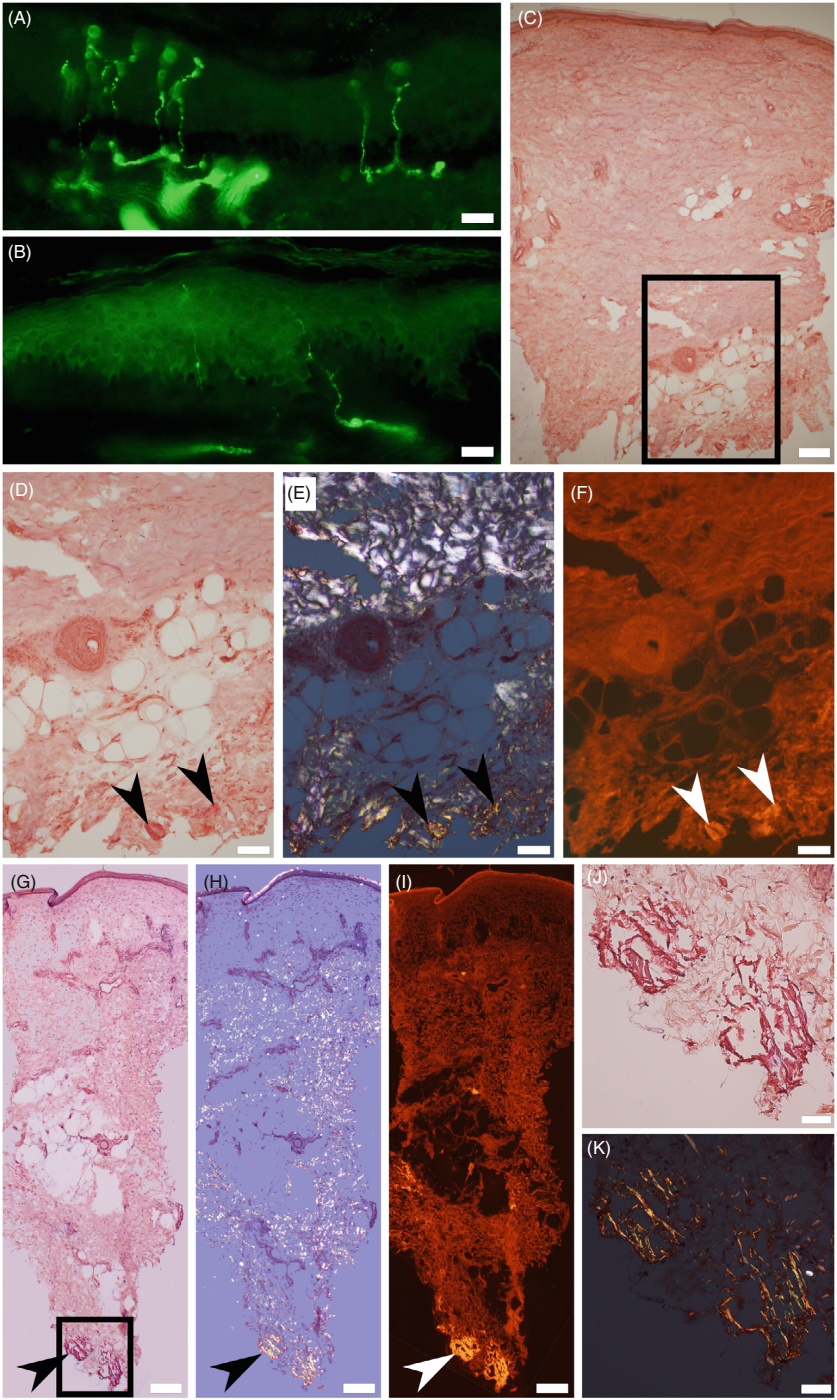
Skin denervation and amyloid in the deep dermis of skin biopsies. Immunofluorescent staining of PGP9.5 shows normal IENFD in a healthy volunteer (A) and low IENFD in a patient without clinical complaints, case 1 (B). Amyloid deposits visualized by Congo red stain in the deep dermis of case 1 (C–F) and in another patient which had no clinical symptoms, case 6 (G–K). Congo red–positive deposits (arrowheads) of amyloid by light microscopy (C, D, G, and J) are birefringent under polarized light (E, H, and K), and enhanced by red spectral fluorescence (F and I). Inset in C refers to D–F, and inset in G to J and K. Scale bars: A and B, 20 μm; C, G, H, and I, 200 μm; D–F, 100 μm; J and K, 50 μm.

Congo red staining showed histological evidence for amyloidosis which was situated in the deep dermis in all of the 16 patients that underwent skin biopsy. These included two patients (cases 1 and 6) that had no clinical complaints but did show evidence for a median neuropathy at the wrist, polyneuropathy, and in one a cardiomyopathy (Fig. 1). In 9/11 (82%) cases two samples were available, with amyloid present in only one, indicating the patchy distribution of these deposits. In two patients an endocardial biopsy was also obtained and was positive as well. Congo red staining in the deep dermis was detected in 11/11 (100%) amyloid‐related cardiomyopathy patients that had skin biopsies.

In three patients a skin sample was not obtained. One had positive Congo red staining on bone‐marrow biopsy in the absence of hematological disease, and in another patient amyloid was detected on rectal biopsy.

### Cardiac manifestations

Dyspnea on exertion early in the course of the disease was reported by only 2/19 (11%) patients. In one, this was related to ischemic heart disease. Palpitation and syncope were not reported as early symptoms. Cardiomyopathy consistent with cardiac amyloidosis was present in 16/19 (84%) patients (Table 2). The mean age of cardiomyopathy diagnosis was 63 ± 5.0 (range 52–70). In two cases, cardiomyopathy diagnosis preceded neuropathy symptoms (cases 14 and 18). LVH of 12 mm or more per echocardiography was found in 15/16 (94%) cardiomyopathy patients at the mean age of 61 ± 4.7 years (range 50–69). Only one patient had heart failure in the absence of LVH (case 18). In that case scintigraphy was negative as well (Perugini grade 1) but CMR showed highly suggestive evidence for amyloid cardiomyopathy. Left ventricular ejection fraction (LVEF) ranged between 30% to 60%. Only 5/19 (26%) patients had reduced systolic function (LVEF<50%). Left ventricular global longitudinal strain (GLS) was studied in 13 patients with cardiomyopathy and was reduced in all of them (ranging −6.9 to −14.4; absolute normal value ≥15). Strain pattern was typical or compatible with apical sparing in 11/14 (79%) patients.

CMR was available in 8 patients, of which 7 had LVH and one with heart failure. All had extensive late enhancement with gadolinium distributed with a noncoronary transmural or sub‐endocardial pattern.

**Figure 2:**
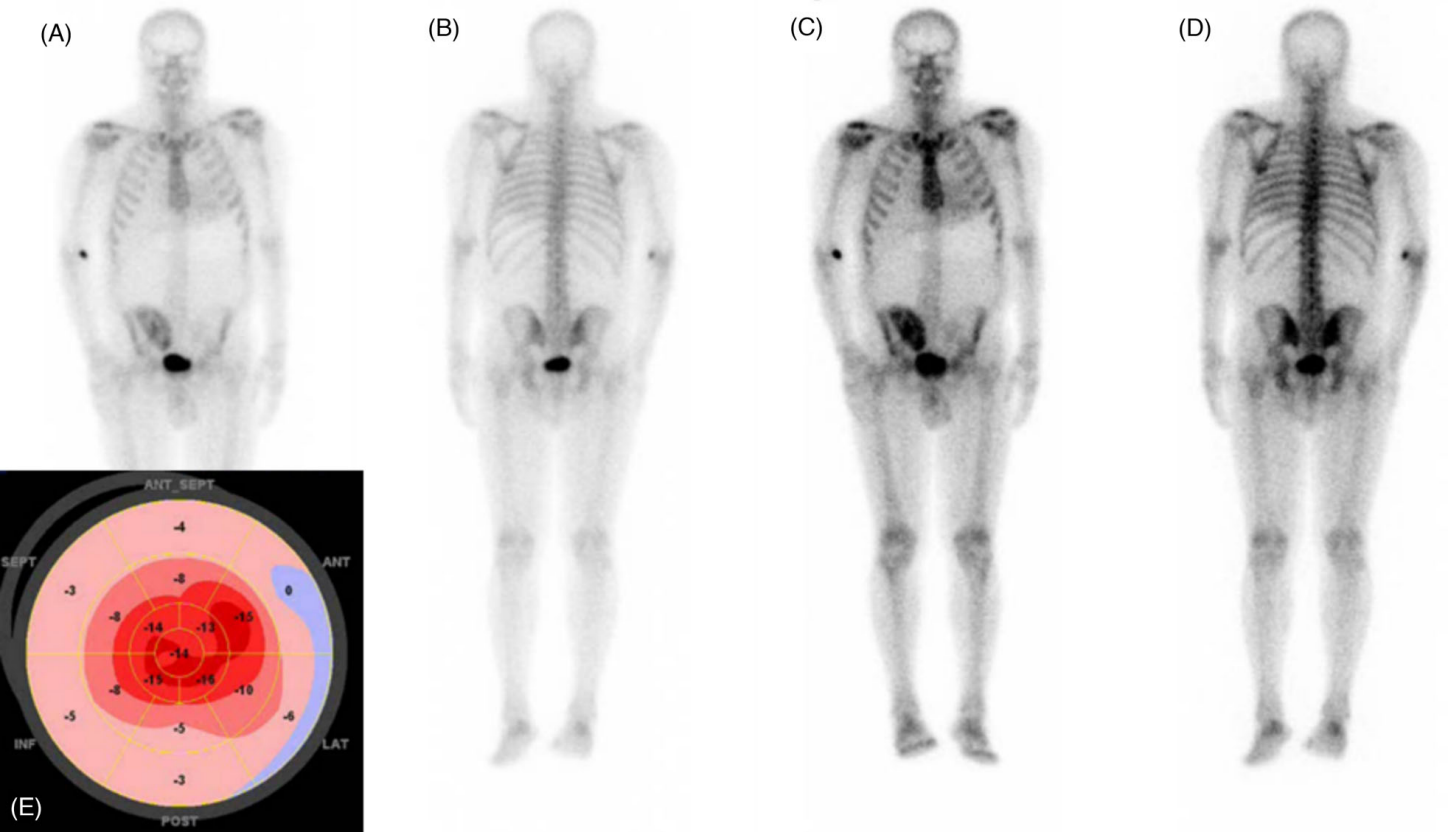
False negative ^99m^Tc‐DPD bone scan images (Perugini grade 1) in a 67 y.o. patient with ATTRS77Y cardiomyopathy (case 3). Anterior (A and C) and posterior (B and D) body images of early (A and B) and delayed (3 h; C and D) scans are shown. At the time of evaluation, the patient had NYHA III heart failure. Maximal left ventricular wall thickness was 15 mm by echocardiography and 25 mm by cardiac MR. The insert (E) illustrates reduced left ventricular longitudinal strain (average − 8) with apical sparing which is typical for amyloidosis. Note uptake in transplanted right kidney.

Scintigraphy with ^99m^Tc‐PYP or ^99m^Tc‐DPD was performed in 9 patients. All of them showed characteristic amyloid cardiomyopathy per echocardiography GLS and/or CMR. Scintigraphy was positive (Perugini grade ≥2) in only 2/9 (22%), which both employed ^99m^Tc‐PYP (Table 2 and Fig. 2).

### Time to diagnosis, treatment and survival

The diagnosis of ATTRS77Y was established 5 ± 4.2 years (range 0–12) following initial neurological or cardiac symptoms.

Seventeen patients were treated with Tafamidis and/or Patisiran. Eleven patients received Tafamidis 20 mg, and two Tafamidis 61 mg (due to cardiomyopathy). After Patisiran became available in Israel, 10 were treated with this agent (7 of them were switched from Tafamidis to Patisiran).

One patient who was treated with liver transplantation at age 62 died due to amyloid‐related cardiac complications at the age of 70, 13 years following symptom onset. Four patients which were not treated by liver transplantation died due to amyloidosis‐related cardiac complications. The mean age at death was 70 ± 3.7 years (range 64–72), 2–13 years after sensory symptoms onset (average 9 ± 4.7). Two of these patients received no disease‐modifying treatment, since at that time treatment was unavailable, or not indicated due to a high disease score. One patient started Tafamidis treatment 8 years after symptom onset and died 2 years later, prior to the availability of Patisiran. Another patient started Tafamidis treatment 10 years after symptoms' onset, switched to treatment with Patisiran 1 year later due to disease progression, and died 16 months later due to amyloidosis‐related cardiac complications.

### Characterization of amyloid‐negative Ser77Tyr mutation carriers

#### Clinical, electrophysiological, and skin biopsy findings

Additional 37 individuals with the Ser77Tyr mutation at a heterozygous state were identified, of whom 30 (15 males) had evaluation for the presence of amyloid disease (Tables 3 and 4). Skin biopsy, employing 2 samples from each subject, accordingly showed no evidence for amyloid in any of them. NYHA score of all was 1, and echocardiography excluded LVH in 13/14 carriers, at the age of 46 ± 9.4 years (range 32–64; Table 4). Scintigraphy was performed by 4, and CMR by 3, including a 64‐year‐old female that had both exams, which were normal. One individual (case 11) had interventricular septum thickness of 12 mm, but his CMR was negative.

**Table 3:** Amyloid‐negative mutation carriers' characteristics: demographics, clinical symptoms, neurological scores, electrodiagnostic and biopsy findings.

Case	Sex	Family	Additional disorders	Symptoms				Exam			Electrophysiological studies				Skin biopsy	
				UE Numb/tingling/pain	LE Numb/tingling/pain	Erectile dysfunction	Urinary incontinence	Age at evaluation (y)	MRC score	NIS‐LL	Median neuropathy: Bilateral	Median neuropathy: Unilateral	Polyneuropathy	Abnormal SSR	Age at evaluation (y)	IENFD <5th percentile
1	F	C		−	−	NA	−	27	60	0	−	−	−	−	27	+
2	F	B		−	−	NA	−	28	60	0	−	−	−	−	28	+
3	M	C		−	−	−	−	31	60	0	−	−	−	−	31	−
4	M	C		−	−	−	−	32	60	0	−	−	−	+	32	+
5	M	B		−	−	−	−	32	60	0	−	−	−	−	32	+
6	M	C		−	−	−	−	32	60	0	−	−	−	−	32	+
7	F	A		−	−	NA	−	34	60	0	−	−	−	+	34	+
8	M	C		−	−	−	−	37	60	2	−	−	−	−	37	−
9	F	D		−	−	NA	−	37	60	0	+	−	−	−	37	−
10	M	E		−	−	−	−	41	60	4	+	−	−	−	41	−
11	M	D	HTN	−	−	−	−	42	60	0	−	−	−	+	42	+
12	F	A		−	−	NA	−	48	60	0	−	−	−	−	48	−
13	F	C		−	−	NA	−	61	60	0	−	−	−	−	61	−
14	F	E		+	+	NA	−	22	60	2	−	−	−	−	22	+
15	M	C		+	−	−	−	28	60	6	−	−	−	−	28	+
16	M	E		+	−	−	−	32	60	2	−	−	−	−	32	−
17	F	C		+	+	NA	−	33	60	0	−	+	−	−	33	+
18	F	C		+	−	NA	−	34	60	1	−	−	−	−	34	−
19	M	D		+	+	−	−	39	60	0	−	−	−	−	39	−
20	M	D		+	+	−	−	42	60	0	−	−	−	−	42	+
21	M	C		+	−	−	−	42	60	0	−	−	−	−	42	−
22	M	E		+	−	−	−	45	60	2	−	−	−	−	45	−
23	M	C		+	−	−	−	45	60	0	+	−	−	−	45	−
24	F	D		+	−	NA	−	45	59	4	−	−	−	−	45	+
25	F	D		+	+	NA	−	47	60	6	−	+	−	−	47	+
26	F	A		−	−	NA	+	49	60	0	−	−	−	−	48	+
27	F	E	HG	+	+	NA	−	45	60	2	−	−	−	−	48	+
28	F	D		+	−	NA	−	50	60	6	−	−	−	−	49	+
29	M	C	T2D	+	−	+	−	54	60	3	+	−	−	+	54	−
30	F	C	HG	+	+	NA		62	59	12	+	−	−	+	63	+

F, female; HG, hyperglycemia, HTN, arterial hypertension; IENFD, intraepidermal nerve fiber density percentile; LE, lower extremity; M, male; MRC, medical research council; NA, not applicable/applied; NIS‐LL, neurology impairment score of lower limb; SSR, sympathetic skin response; UE, upper extremity; SSR, sympathetic skin response; T2D, type 2 diabetes; y, years.

**Table 4:** Amyloid‐negative mutation carriers' cardiac evaluation findings.

Case	Sex	Family	Age at evaluation (y)	Electrocardiogram		Echocardiography					CMR	Scintigraphy	
				Conduction	Arrhythmia	IVS (mm)	PWD (mm)	LVEF (%)	GLS	GLS pattern	Findings	Tracer	Perugini grade
7	F	A	34	NL	‐	9	9	60					
9	F	D	38	Short PR	‐	8	8	58	−18.3	NL			
11	M	D	42	NL	‐	12	9	60	−20.3	NL	NL		
12	F	A	49	NL	‐	9	7	60	−31	NL			
13	F	C	59	NL	‐	9	9	60					
16	M	E	32	NL	‐	10	9	60					
17	F	C	33	NL	‐	9	9	55					
24	F	D	45	Short PR	‐	7	6	60	−21	NL			
25	F	D	47	NL	‐	9	8	60	−20.3	NL			
26	F	A	49	NL	‐	10	9	60	−18	Non specific		DPD	0
27	F	E	48	NL	‐	8	6	60			NL	DPD	0
28	F	D	49	ICRBBB, Lt axis	‐	8	6	60	−19.6	NL			
29	M	C	52	NL	‐	10	10	70	−21.6	NL		PYP	0
30	F	C	64	NL	‐	9	8	60	−18	NL	NL	PYP	0

CMR, cardiac magnetic resonance; DPD, ^99m^Tc‐3,3‐diphosphono‐1,2‐propano‐ dicarboxylic acid; F, female; GLS, global longitudinal left ventricular strain; ICRBBB, incomplete right bundle branch block; IVS, Inter‐ventricular septum;  LVEF, left ventricular ejection fraction; Lt, left; M, male; NL, normal; PWD, Posterior wall diameter; PYP, ^99m^Tc‐pyrophosphate; y, years.

**Figure 3:**
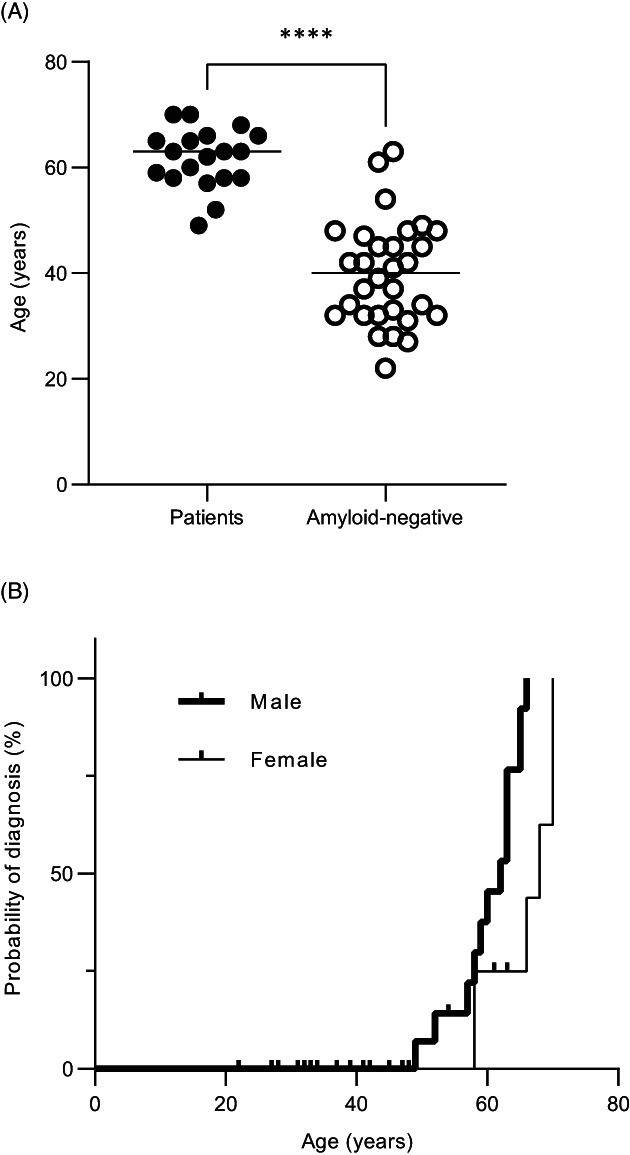
(A) Age distribution of patients at diagnosis and amyloid‐negative carriers at evaluation. (B) Survival analysis for predicting age at diagnosis. Males (*n* = 13) were diagnosed as affected earlier, at a median onset of 62 years, while women (*n* = 6) were at 68 years (*p* = 0.0076). ****p < 0.0001.

Most amyloid‐negative carriers were younger than patients (40 ± 9.8 years, range 22–63 vs. 62 ± 5.7, range 49–70; *p* < 0.0001; Fig. 3A). More than half of the amyloid‐negative carriers (17/30; 57%) complained of various intermittent or constant neuropathic pain in the upper and/or lower extremities. In 3 of them, these were possibly related to T2D or hyperglycemia but in the reminder, no relevant medical or laboratory finding was identified. Although the mean age of amyloid‐negative carriers with neuropathy symptoms was older than that of those without symptoms, this was not statistically significant (42 ± 10.0 vs. 37 ± 9.3 years, respectively; *p* = 0.165).

A median neuropathy at the wrist was identified in 7/30 (23%) amyloid‐negative carriers, 5 in the group with and 2 in those without neuropathy symptoms (Table 3). This was bilateral in 3 cases in the group with and in 2 in the group without symptoms. NCSs showed no evidence for large‐fiber polyneuropathy and EMG was normal in all. The SSR was absent at the foot in 5/30 (17%) of amyloid‐negative carriers, of whom 2 had T2D or hyperglycemia.

Skin biopsy showed an abnormally low IENFD in 17/30 (57%) of amyloid‐negative carriers; in 10/17 (59%) with and in 7/13 (54%) without neuropathy‐related symptoms, but this difference was not significant.

Seven carriers did not complete the required evaluation, and therefore were excluded from the analysis. Their mean age at clinical evaluation was 40 ± 11.9 years (range 25–56). Upper and/or lower limb sensory symptoms were reported by 3 of them, and clinical exam was normal in all. Electrodiagnostic studies were performed by 4 and echocardiography by 3, and were all normal. One 48‐year‐old female had a skin biopsy which showed epidermal denervation but no amyloid, and was reluctant to performing cardiac imaging. These findings, despite the incomplete data, are similar to that of the reminder of unaffected carriers.

### Risk for disease manifestation

The risk of becoming affected by (i.e. diagnosed with) amyloidosis increased with age, as most of the patients were older than 50 years (18/19; 95%), while most of the amyloid‐negative carriers were younger than that (24/30; 80%). We aggregated the data of patients and amyloid‐negative carriers that were older versus younger than 50 and found a significant risk for becoming affected above this age (*p* < 0.001). At the age of 49, 4.4% of carriers become affected by amyloidosis, and 100% at the age of 70, with a median at 63 years. Male carriers were diagnosed with amyloidosis at an earlier age in comparison to females (Log‐rank Mantel‐Cox test *p* = 0.0076; Fig. 3B).

## Discussion

We identified the presence of the *TTR* Ser77Tyr mutation in 5 families of Jewish Yemenite origin. Occurrence of this probable founder mutation in this population enabled testing of family members at risk and identification of undiagnosed patients as well as amyloid‐negative carriers. Thus, our study provides a valuable opportunity to describe features of ATTRS77Y.

Symptoms related to CTS were the initial manifestation of amyloidosis in most patients, presented during their 50 s. Erectile dysfunction commonly occurred earlier in males. Electrodiagnostic studies near diagnosis showed evidence for a median neuropathy at the wrist in almost all patients, commonly bilateral, and large‐fiber polyneuropathy in about two‐thirds. However, most patients had additional systemic disorders such as T2D or renal failure that likely contributed to nerve damage in these individuals. Therefore, the sensorimotor and autonomic symptoms in patients with combined systemic disorders probably resulted from both etiologies, though that due to amyloidosis is considered to be more aggressive. The SSR which tests autonomic small fibers did not correlate with small fiber loss, which was evident on skin biopsy in almost all patients. Interestingy, cardiomyopathy was identified in most patients by the time polyneuropathy was present. Amyloid deposits were detected in the deep dermis in all cardiomyopathy patients which had a skin biopsy.

The Ser77Tyr mutation was previously reported in Jewish Yemenite descents in Israel^10^, ^11^ as well as in other populations worldwide.^31^, ^32^, ^33^ In general, our findings are consistent with previous studies which identified early CTS, evolving polyneuropathy and restrictive cardiomyopathy. Nevertheless, we provide the frequency of clinical symptoms and signs, as well as features obtained by using common diagnostic tools, at the initial course of the disease. Our results highlight that absence of symptoms and signs is not rare and does not rule‐out amyloidosis. In particular, at the initial course, about 10% of patients have no clinical complaints, bilateral and even unilateral median neuropathy at wrist is not present in all patients, electrodiagnostic evidence for large fiber polyneuropathy is absent in a third and skin biopsy indication for small fiber neuropathy may be absent as well. Furthermore, cardiomyopathy symptoms may precede peripheral neuropathy symptoms, and substantial cardiac disease may be undetectable by scintigraphy.

Our approach for histopathological diagnosis was based on skin biopsy from the ankle. The sensitivity of a 3 mm skin biopsy from this region was reported to be 70% for detecting amyloid in ATTRv polyneuropathy,^34^ increasing from 25% in early disease to 100% in the late stage of the disease.^35^ The likelihood of amyloid detection was shown to increase from 74% with a single skin biopsy from the ankle to 86% with an additional skin biopsy from the thigh,^13^ and was highest at the ankle region.^12^ This is comparable to the diagnostic sensitivity reported for Sural nerve (up to 83%),^36^, ^37^ salivary gland (75–91%),^38^, ^39^ and fat pad (83%)^40^ biopsies.

A major advantage of this study is the comprehensive phenotyping, including neurological examination, electrophysiology, biopsy and echocardiography in a single referral clinic. Systematic evaluation and differentiation between patients and amyloid‐negative carriers allowed the calculation of ATTRS77Y penetrance. Accordingly, as the risk for becoming affected by amyloidosis increased following age 50 and initial symptoms preceded diagnosis by a few years, we estimate that as the predicated age of disease onset. In contrast to previous reports about Ser77Tyr,^41^ we found that all carriers eventually become ill at the end of their seventh decade, with males becoming affected earlier than females. In addition, while the number of males and females in the amyloid‐negative carrier group was similar, males were the majority within the patients' group. This corroborates previous reports of males more commonly affected than females by the predominantly cardiac, mixed and neurological phenotypes of ATTRv, in patients with the Ser77Tyr as well as with other mutations.^42^ It is worth noting in this context, that the early occurrence of erectile dysfunction did not contribute to early evaluation of amyloidosis.

Nonspecific neuropathic symptoms were common in amyloid‐negative carriers, and in some cases were noticed many years prior to the predicted age of disease onset. Most of these subjects showed skin denervation, as was previously reported in presymptomatic carriers.^12^, ^34^, ^43^ However, the absence of amyloid in the skin and lack of heart involvement declined amyloidosis diagnosis and treatment. Polyneuropathy is common in the general population, affecting 1.2–2.4% of individuals between 50–60 years of age, and increases with age.^44^ Similarly, the prevalence of small‐fiber polyneuropathy^45^ is much higher than that of ATTRS77Y. Therefore, an isolated finding of skin denervation in mutation carriers cannot be regarded as a hallmark of amyloidosis. Nevertheless, the high occurrence of skin denervation in amyloid‐negative carriers raises concern that current diagnostic techniques, which depend on detection of amyloid in tissue or amyloid‐related cardiac imaging, are not sensitive enough to provide evidence for early TTR‐induced pathology. Indeed, nonfibrillar oligomer deposits in tissue accumulate years prior to symptomatic disease onset,^28^, ^46^ and have shown toxicity against Schwannoma cell lines *in vitro* and in sural nerve biopsies, prior to formation of mature amyloid fibrils.^46^, ^47^ Hence, neuropathy‐related symptoms in our amyloid‐negative carriers may be due to a nonfibrillary deposition process.

Similarly, we show that CTS or asymptomatic median neuropathy at the wrist occurs early in ATTRS77Y amyloidosis, consistent with previous reports.^48^, ^49^ CTS is a common condition in the general population affecting approximately 3–6% of adults.^50^ Furthermore, it is most common at ages 40–49 years,^51^ which approaches the predicted age of ATTR onset due to the Ser77Tyr and other *TTR* mutations. Our findings of a median neuropathy in 23% of amyloid‐negative carriers suggest again a pre‐amyloid active disease, but further studies are required to establish this.

Scintigraphy is a mainstay of contemporary ATTR cardiomyopathy diagnosis. Several studies indicated that scintigraphy with ^99m^Tc‐DPD and ^99m^Tc‐PYP has high sensitivity and specificity for cardiac ATTR diagnosis.^15^, ^52^, ^53^ Furthermore, this test effectively differentiates cardiac ATTRv from AL amyloidosis in the absence of a monoclonal protein and serum free light chain abnormality, thus reducing the need for endomyocardial biopsy and typing of the amyloid fibrils.^14^ However, the feasibility and safety of repeated scintigraphy through a long‐term follow‐up of *TTR* mutation carriers has not been established, thus favouring periodical echocardiography and in selected cases CMR as the main repeated screening modalities. In our experience, scintigraphy was of low yield as it was normal or nondiagnostic in 7/9 of our patients. Some of these had severe cardiomyopathy per other imaging modalities. This observation is of importance, because a negative scintigraphy study may mistakenly defer TTR‐amyloidosis diagnosis, and delay treatment. This is consistent with a previous report of 3 patients with ATTRS77Y cardiomyopathy which showed grade 0 or grade 1 ^99m^Tc‐DPD uptake in spite of typical features of cardiac amyloidosis on echocardiogram and CMR.^16^ Similarly, low sensitivity of scintigraphy was reported with the Phe64Leu *TTR* mutation^17^ and ^99m^Tc‐DPD scintigraphy had suboptimal sensitivity to detect cardiac involvement in ATTRV30M.^54^ Accordingly, we used CMR to validate or exclude the diagnosis of amyloid cardiomyopathy in selected cases. We did not employ alternative methods for cardiac evaluation, such as the amino‐terminal prohormone of brain natriuretic peptide (NT‐proBNP) which is increased in plasma even in the early stages of cardiac amyloid infiltration, in correlation with systolic dysfunction and/or LVH.^55^ We did however systematically search for LVH in carriers approaching the age of disease onset.

Several limitations of this study should be acknowledged. In this retrospective analysis we identified the age of symptom onset and age at diagnosis but could not accurately determine the age of disease onset. This requires a prospective long term follow‐up with periodic testing to assess neurologic and cardiac disease progression, which is currently ongoing. We did not perform uniform cardiac evaluation that includes scintigraphy and CMR for some of the patients, as well as echocardiography for all amyloid‐negative carriers. CMR may have shown heart involvement in amyloid‐negative carriers and alternate their status to patients. We also did not systematically evaluate the effect of the novel drugs (Tafamidis and Patisiran) over time, and this will be hopefully addressed in the future. In addition, the possible involvement of other body systems or organs (for example ophthalmological or autonomic) was not consistently investigated. Last, seven mutation carriers did not complete the required evaluation and were excluded from the analysis.

To conclude, the *TTR* Ser77Tyr mutation causes a rare disease with typical but common presenting symptoms, usually due to a median and polyneuropathy, and cardiomyopathy. Identification of these in a mutation carrier, with or without clinical complaints and findings, suggests amyloidosis, particularly after the age of 50 years. This may be confirmed by Congo red staining of tissue, readily from skin, or detection of heart involvement by echocardiography.

## Author Contributions

Amir Dori, Michael Arad and Lior Greenbaum contributed to the conception and design of the study; Amir Dori, Michael Arad, Yishay Wasserstrum, Arthur Pollak, Vera Nikitin, Merav Ben‐David, Jana Shamash, Ayelet Hashachar Nahum, Efrat Shavit‐Stein, Liran Domachevsky, Rafael Kuperstein, Dan Dominissini, Natalia Shelestovich, Menachem Sadeh, Elon Pras and Lior Greenbaum, contributed to the acquisition and analysis of data; Amir Dori, Michael Arad, and Lior Greenbaum contributed to drafting a significant portion of the manuscript or figures.

## Funding Information

Detection of TTR mutations in Israel was supported by a research grant from Pfizer [#59354385].

## Conflict of Interest

This is to acknowledge that A. Dori received consultancy fees, honoraria for lectures and travel meeting attendance support from Pfizer, and investigator‐initiated research grant support from Pfizer and Alnylam Pharmaceuticals industry (companies that manufacture the drugs described is in this study). Additionally, A. Dori is the local site's primary investigator in Pfizer's Transthyretin Amyloidosis Outcome Survey (THAOS) and Alnylam's Global Observational Study of Patients with Transthyretin‐Mediated Amyloidosis (ConTTRibute) study. M. Arad received lectures honoraria and fees for participation in an advisory board from Pfizer.

## Supporting information


Table S1

Table S2
