# Supplementary material for: Ser77Tyr transthyretin amyloidosis in Israel: Initial manifestations and diagnostic features
Source: Ann Clin Transl Neurol. 2023 Feb 11;10(4):553–67. doi: 10.1002/acn3.51741 (PMC10109316; doi:10.1002/acn3.51741)
Supplement: Supplementary file 1 — Table S1 [file ACN3-10-553-s001.docx]

|  |  | Median | | | | Ulnar | | | | | Tibial | | | | Peroneal | | | | |
| --- | --- | --- | --- | --- | --- | --- | --- | --- | --- | --- | --- | --- | --- | --- | --- | --- | --- | --- | --- |
| Case | NL  Side | Dist  Lat  <4.4 ms | FA CV  >49 m/s | Dist Amp  >4 mV | F-lat  >32 ms | Dist Lat  <3.5 ms | FA CV  >49 m/s | Elb CV  >49 m/s | Dist Amp  >6  mV | F-lat  >32 ms | Dist Lat  <6 ms | FL CV  >39 m/s | Dist Amp  >3  mV | F-lat  >52 ms | Dist  Lat  <6 ms | FL CV  >39 m/s | Knee CV  >39 m/s | Dist Amp  >2 mV | F-lat  >52 ms |
| 1 | R | 4.4 | 53 | 9.3 | 31 | 2.3 | 55 | 74 | 6.1 | 28 | nt | nt | nt | nt | nt | nt | nt | nt | nt |
|  | L | 3.6 | 51 | 12 | 28 | 2.4 | 63 | 66 | 6.6 | 28 | 3.4 | 49 | 4.8 | 55 | 4.6 | 50 | 43 | 4.7 | 54 |
| 2 | R | 7.8 | 21 | 1.1 | NR | 3.3 | 48 | 35 | 1.0 | nt | nt | nt | nt | nt | nt | nt | nt | nt | nt |
|  | L | 7.4 | 38 | 0.6 | nt | 4.9 | 55 | 39 | 0.8 | nt | 5.1 | 38 | 0.3 | nt | 3.0 | 38 | 36 | 0.4 | nt |
| 3 | R | 4.1 | 44 | 5.5 | 32 | 3.0 | 51 | 55 | 4.8 | 32 | 4.8 | 40 | 3.2 | 40 | 5.2 | 40 | 46 | 1.0 | 55 |
|  | L | 4.0 | 45 | 4.1 | 31 | 3.2 | 55 | 51 | 5.0 | 31 | nt | nt | nt | nt | nt | nt | nt | nt | nt |
| 4 | R | 7.1 | 42 | 2.5 | 35 | 3.5 | 53 | 35 | 6.1 | 34 | 4.2 | 40 | 2.5 | 58 | 5.4 | 43 | 41 | 2.2 | 53 |
|  | L | 5.4 | 50 | 7.2 | 34 | 3.8 | 51 | 38 | 4.7 | 33 | nt | nt | nt | nt | nt | nt | nt | nt | nt |
| 5 | R | 4.8 | 45 | 6.2 | 33 | 3.7 | 51 | 58 | 7.2 | 32 | nt | nt | nt | nt | nt | nt | nt | nt | nt |
|  | L | 5.2 | 45 | 5.1 | 39 | 3.8 | 50 | 56 | 6.1 | 33 | 4.3 | 32 | 1.6 | 35 | 5.7 | 33 | 33 | 2.1 | NR |
| 6 | R | 3.5 | 53 | 8.5 | 33 | 2.7 | 57 | 52 | 8.8 | 32 | nt | nt | nt | nt | nt | nt | nt | nt | nt |
|  | L | 4.4 | 52 | 7.6 | 31 | 3.1 | 55 | 41 | 9.3 | 32 | 3.8 | 44 | 4.9 | 55 | 4.6 | 41 | 41 | 1.9 | 57 |
| 7 | R | 3.5 | 58 | 7.0 | 26 | 2.1 | 55 | 69 | 8.8 | 25 | 3.1 | 55 | 6.5 | 44 | 3.4 | 55 | 55 | 6.8 | 39 |
|  | L | 3.6 | 59 | 13 | 27 | 2.3 | 63 | 69 | 6.4 | 25 | 3.4 | 55 | 12 | 41 | 4.0 | 63 | 49 | 6.6 | 41 |
| 8 | R | 4.7 | nt | 0.5 | nt | 2.6 | 50 | 42 | 3.0 | 38 | 4.3 | 36 | 1.3 | 59 | 4.8 | 36 | nt | 0.6 | nt |
|  | L | NR | NR | NR | NR | 2.7 | 53 | 39 | 4.8 | 31 | nt | nt | nt | nt | nt | nt | nt | nt | nt |
| 9 | R | 4.8 | 51 | 5.8 | 31 | 2.7 | 51 | 37 | 9.3 | 33 | 3.3 | 42 | 3.3 | 62 | 4.0 | 46 | 48 | 11 | 54 |
|  | L | 5.4 | 51 | 4.2 | 32 | 2.8 | 64 | 41 | 8.3 | 27 | 2.2 | 46 | 2.2 | 65 | 4.4 | 48 | 45 | 7 | 55 |
| 10 | R | NR | NR | NR | nt | NR | NR | NR | NR | nt | nt | nt | nt | nt | nt | nt | nt | nt | nt |
|  | L | 4.3 | 32 | 0.2 | nt | 3.1 | 32 | 30 | 0.9 | 59 | NR | NR | NR | nt | nt | NR | NR | NR | nt |
| 11 | R | 5.7 | 37 | 4.9 | 35 | 2.9 | 48 | 49 | 5.9 | 29 | 5.3 | 45 | 0.3 | nt | 5.0 | 46 | 41 | 2.4 | nt |
|  | L | 4.5 | 43 | 6.3 | 32 | 3.1 | 58 | 48 | 9.5 | 29 | nt | nt | nt | nt | nt | nt | nt | nt | nt |
| 12 | R | 3.5 | 51 | 5.7 | 27 | 2.7 | 56 | 45 | 7.6 | 32 | 4.5 | 43 | 5.2 | 56 | 4.4 | 43 | 46 | 4.2 | 43 |
|  | L | 3.6 | 50 | 5.9 | 32 | 2.6 | 52 | 55 | 6.6 | 31 | 4.5 | 41 | 4.4 | 51 | 4.6 | 50 | 48 | 6.3 | 50 |
| 13 | R | 5.6 | MGA | 9.4 | 28 | 2.1 | 61 | 77 | 10 | 26 | nt | nt | nt | nt | nt | nt | nt | nt | nt |
|  | L | 4.9 | MGA | 6.4 | 28 | 2.2 | 73 | 77 | 11 | 26 | 3.5 | 40 | 11 | 48 | 3.1 | 54 | 77 | 19 | 42 |
| 14 | R | 2.8 | 54 | 11 | 25 | 2.0 | 63 | 66 | 12 | 28 | nt | nt | nt | nt | nt | nt | nt | nt | nt |
|  | L | 2.7 | 56 | 12 | 25 | 2.2 | 69 | 58 | 8.1 | 27 | 3.2 | 52 | 8.5 | 49 | 5.6 | 45 | 47 | 0.9 | nt |
| 15 | R | 4.4 | 46 | 5.0 | 35 | 3.2 | 47 | 30 | 4.1 | 38 | 5.1 | 39 | 4.0 | 60 | 5.1 | 47 | 47 | 3.5 | 56 |
|  | L | 4.1 | 50 | 5.2 | 34 | 3.2 | 49 | 32 | 6.3 | 37 | 3.9 | 43 | 3.0 | 60 | 5.2 | 43 | 47 | 5.1 | 59 |
| 16 | R | 4.9 | 50 | 5.0 | 30 | 3.4 | 53 | 46 | 5.9 | 32 | 5.1 | 49 | 0.3 | nt | 5.5 | 38 | 36 | 1.3 | nt |
|  | L | 4.3 | 51 | 5.6 | 31 | 3.1 | 53 | 45 | 4.0 | 31 | nt | nt | nt | nt | nt | nt | nt | nt | nt |
| 17 | R | 4.3 | 34 | 0.8 | nt | 3.4 | 65 | 40 | 3.1 | 27 | 5.1 | 39 | 0.8 | nt | 5.5 | 55 | 53 | 0.5 | nt |
|  | L | 4.9 | 43 | 1.2 | 25 | 3.5 | 63 | 34 | 2.8 | 34 | 5.3 | 42 | 0.9 | nt | 6.3 | 44 | 45 | 0.5 | nt |
| 18 | R | nt | nt | nt | nt | nt | nt | nt | nt | nt | Prol | Slow | Small | nt | Prol | Slow | Slow | Small | nt |
|  | L | NL | NL | NL | NL | NL | NL | Slow | NL | NL | Prol | Slow | Small | NL | Prol | Slow | Slow | Small | nt |
| 19 | R | 4.0 | 58 | 6.4 | 26 | 2.3 | 58 | 44 | 6.8 | 25 | 5.4 | 50 | 0.7 | nt | 4.7 | NR | NR | 0.2 | nt |
|  | L | 4.2 | 53 | 5.5 | 27 | nt | nt | nt | nt | nt | nt | nt | nt | nt | nt | nt | nt | nt | nt |

Legend: Motor nerve conduction studies.

Amp, amplitude; CV, conduction velocity; Dis, Distal; Elb, Elbow; FA, forearm; FL, Foreleg; L, left; Lat, Latency; NL, normal; No response; nt, not tested; MGA, Martin-Gruber anastomosis; m/s meters pre-second; ms, milliseconds; mV, millivolt; Prol, Prolonged; R, right. For patient #18 only descriptive data was available.
