# Supplementary material for: Ser77Tyr transthyretin amyloidosis in Israel: Initial manifestations and diagnostic features
Source: Ann Clin Transl Neurol. 2023 Feb 11;10(4):553–67. doi: 10.1002/acn3.51741 (PMC10109316; doi:10.1002/acn3.51741)
Supplement: Supplementary file 2 — Table S2 [file ACN3-10-553-s002.docx]

|  |  | Median | | Ulnar | | Radial | | Sural | | Peroneal | |
| --- | --- | --- | --- | --- | --- | --- | --- | --- | --- | --- | --- |
| Case | NL  Side | Lat  <3.5 ms | Amp  >15 uV | Lat  <3.1 ms | Amp  >10  uV | Lat  <2.9 ms | Amp  >15 uV | Lat  <3.8 ms | Amp  >5 uV | Lat  <3.4 ms | Amp  >2 uV |
| 1 | R | 3.5 | 25 | 2.4 | 25 | 1.9 | 19 | 2.7 | 15 | 3.4 | 9 |
|  | L | 3.3 | 33 | 2.7 | 19 | 1.8 | 26 | 2.9 | 12 | 3.4 | 9 |
| 2 | R | NR | NR | NR | NR | NR | NR | NR | NR | NR | NR |
|  | L | NR | NR | NR | NR | NR | NR | NR | NR | NR | NR |
| 3 | R | NR | NR | 3.0 | 5.3 | 2.4 | 7.1 | NR | NR | NR | NR |
|  | L | 3.8 | 3.2 | 3.2 | 7.5 | 2.3 | 7.1 | NR | NR | NR | NR |
| 4 | R | NR | NR | 3.3 | 4.4 | 2.7 | 4 | NR | NR | NR | NR |
|  | L | 4.6 | 4.1 | 3.4 | 3.1 | 3.4 | 3 | NR | NR | NR | NR |
| 5 | R | 4.8 | 2.8 | 3.3 | 9.7 | 2.3 | 4.4 | NR | NR | NR | NR |
|  | L | 5.1 | 1.8 | 3.7 | 8.1 | 2.6 | 5.6 | NR | NR | NR | NR |
| 6 | R | 3.5 | 16 | 2.6 | 22 | 2.2 | 22 | 3.3 | 5.5 | 3.5 | 4.4 |
|  | L | 4.2 | 18 | 2.9 | 11 | 2.4 | 20 | 2.6 | 3.4 | 3.2 | 5.8 |
| 7 | R | 4.3 | 14 | 2.4 | 39 | 1.8 | 52 | 3.1 | 10 | 2.6 | 17 |
|  | L | 4.2 | 30 | 2.7 | 37 | 1.9 | 48 | 2.9 | 13 | 2.8 | 15 |
| 8 | R | NR | NR | 3.4 | 3.5 | 2.4 | 9 | 3.2 | 2.5 | NR | NR |
|  | L | NR | NR | 2.7 | 3.0 | 2.6 | 13 | 3.2 | 1.1 | NR | NR |
| 9 | R | 4.1 | 5.1 | NR | NR | 2.2 | 22 | 2.8 | 11 | 3.7 | 1.8 |
|  | L | 4.3 | 8.3 | 2.8 | 17 | 2.0 | 10 | 2.8 | 12 | 4.0 | 4.1 |
| 10 | R | NR | NR | NR | NR | NR | NR | NR | NR | NR | NR |
|  | L | NR | NR | NR | NR | NR | NR | NR | NR | NR | NR |
| 11 | R | NR | NR | 3.2 | 4.1 | 2.4 | 7.6 | NR | NR | NR | NR |
|  | L | NR | NR | 3.1 | 4.1 | 2.8 | 5.9 | NR | NR | NR | NR |
| 12 | R | 3.8 | 5.9 | 2.9 | 11 | 2.2 | 14 | 2.8 | 5.2 | 3.5 | 5.1 |
|  | L | 3.4 | 11 | 3.2 | 13 | 2.5 | 15 | 2.7 | 6.6 | 3.2 | 6.2 |
| 13 | R | 4.6 | 8.0 | 2.5 | 30 | 2.0 | 48 | 2.7 | 22 | 2.3 | 19 |
|  | L | 3.6 | 29 | 2.4 | 32 | 1.8 | 51 | 2.4 | 17 | 2.6 | 16 |
| 14 | R | 2.8 | 44 | 2.4 | 39 | 2.2 | 48 | 3.5 | 9.7 | 3.8 | 3.9 |
|  | L | 2.6 | 66 | 2.2 | 30 | 1.8 | 60 | 2.6 | 9.0 | 3.6 | 8.7 |
| 15 | R | 4.1 | 2.1 | 3.6 | 3.3 | 2.4 | 11.1 | 3.3 | 4.0 | NR | NR |
|  | L | 3.8 | 3.4 | 3.2 | 3.2 | 2.3 | 6.0 | 2.9 | 6.3 | NR | NR |
| 16 | R | NR | NR | 3.2 | 1.9 | 2.8 | 5.6 | NR | NR | NR | NR |
|  | L | NR | NR | 3.3 | 2.0 | 2.2 | 6.5 | NR | NR | NR | NR |
| 17 | R | NR | NR | NR | NR | NR | NR | NR | NR | NR | NR |
|  | L | NR | NR | NR | NR | NR | NR | NR | NR | NR | NR |
| 18 | R | nt | nt | nt | nt | nt | nt | NL | Small | NR | NR |
|  | L | Prol | Small | NL | NL | NL | NL | NL | Small | NR | NR |
| 19 | R | 3.2 | 10 | 2.3 | 18 | 2.4 | 31 | NR | NR | NR | NR |
|  | L | 3.5 | 14 | nt | nt | nt | nt | NR | NR | NR | NR |

Legend: Motor nerve conduction studies.

Amp, amplitude; L, left; Lat, Latency; NL, normal; NR, No response; m/s meters pre-second; ms, milliseconds; Prol, Prolonged; R, right; uV, microvolt; nt, not tested.
